# Stratified sero-prevalence revealed overall high disease burden of dengue but suboptimal immunity in younger age groups in Pune, India

**DOI:** 10.1371/journal.pntd.0006657

**Published:** 2018-08-06

**Authors:** Akhilesh C. Mishra, Vidya A. Arankalle, Swapnil A. Gadhave, Pritam H. Mahadik, Shubham Shrivastava, Mandar Bhutkar, Varsha M. Vaidya

**Affiliations:** 1 Interactive Research School for Health Affairs (IRSHA), Bharati Vidyapeeth (Deemed to be University), Katraj, Pune, India; 2 Department Community Medicine, Medical College, Bharati Vidyapeeth (Deemed to be University), Katraj, Pune, India; University of Texas Medical Branch, UNITED STATES

## Abstract

**Background:**

In India, dengue disease is emerging as the most important vector borne public health problem due to rapid and unplanned urbanization, high human density and week management of the disease. Clinical cases are grossly underreported and not much information is available on prevalence and incidence of the disease.

**Methodology:**

A cross sectional, stratified, facility based, multistage cluster sampling was conducted between May 4 and June 27, 2017 in Pune city. A total of 1,434 participants were enrolled. The serum samples were tested for detection of historical dengue IgG antibodies by ELISA using the commercial Panbio Dengue IgG Indirect ELISA kit. Anti-dengue IgG-capture Panbio ELISA was used for detection of high titered antibodies to detect recent secondary infection. We used this data to estimate key transmission parameters like force of infection and basic reproductive number. A subset of 120 indirect ELISA positive samples was also tested for Plaque Reduction Neutralizing Antibodies for determining serotype-specific prevalence.

**Findings:**

Overall, 81% participants were infected with dengue virus (DENV) at least once if not more. The positivity was significantly different in different age groups. All the adults above 70 years were positive for DENV antibodies. Over 69% participants were positive for neutralizing antibodies against all 4 serotypes suggesting intense transmission of all DENV serotypes in Pune. Age-specific seroprevalence was consistent with long-term, endemic circulation of DENV. There was an increasing trend with age, from 21.6% among <36 months to 59.4% in age group 10–12 years. We estimate that 8.68% of the susceptible population gets infected by DENV each year resulting into more than 3,00,000 infections and about 47,000 to 59,000 cases per year. This transmission intensity is similar to that reported from other known hyper-endemic settings in Southeast Asia and the Americas but significantly lower than report from Chennai.

**Conclusions:**

Our study suggests that Pune city has high disease burden, all 4 serotypes are circulating, significant spatial heterogeneity in seroprevalence and suboptimal immunity in younger age groups. This would allow informed decisions to be made on management of dengue and introduction of upcoming dengue vaccines in the city.

## Author summary

Dengue disease, transmitted through the bite of DENV infected mosquitoes, is an increasing health problem in the Asian subcontinent, including India. Dengue ranges from mild undifferentiated fever to circulatory shock and potentially death. Clinical disease gives an incomplete picture of the magnitude of dengue, because many infections are asymptomatic. Presence of antibodies to DENV provides evidence of past infection. This study provides the first estimate of the prevalence and incidence of dengue, based on the data collected from a well-designed, comprehensive serosurvey. By studying age–wise antibody prevalence, we estimated the force of DENV infection by applying a catalytic model to our serosurvey data. Over 81% individuals were positive for DENV antibodies suggesting intense DENV transmission in Pune city. We estimate that 8.68% of the susceptible population gets infected by DENV each year resulting into more than 3,00,000 infections and about 47,000 to 59,000 cases per year. The estimated seroprevalence at 9 years age (SP9), taken as benchmark for introduction of Dengvaxia vaccine by WHO, was 54.17% suggesting moderate transmission intensity of dengue, making introduction of the vaccine unsuitable in younger children.

## Introduction

Dengue disease is an important emerging public health problem in countries of tropical and subtropical regions.[1–3] Estimated annual global burden of disease is approximately 390 million infections, 96 million clinical cases, and 20 thousand deaths, with almost 34% of total dengue cases occurring in India.[4] According to recent estimates, 2·9 million dengue episodes and 5906 deaths, with an economic burden of $950 million occur annually in Southeast Asia (SEA) alone.[5] It is known that disease intensity and disease burden is highly variable between different places within a country or region.[6]

In India, dengue is a reportable disease and all confirmed cases are expected to be reported to government of India through NVDCP, Delhi.[7] Recent studies using various models have suggested gross underreporting of dengue cases. It is estimated that each case reported may be multiplied by 200 to get fair estimate.[8,9]

There are 4 antigenically distinct DENV serotypes (DENV 1–4). Dengue can result from infection with any one of four viral serotypes. Infection with one serotype provides long-term protection to that serotype, but not to others. Thus, DENV seropositive individuals could be monotypic due to primary infection or multitypic due to secondary infections. Presence of certain serotypes, including primary infection with DENV-3 from the SEA region and secondary infection with DENV-2, DENV-3, and DENV-4 also from the SEA region, as well as DENV-2 and DENV-3 from non-SEA regions, increased the risk of severe dengue infections.[10] Thus, age specific distribution for different serotypes and their contributions in monotypic and multitypic cases are worthy of special consideration.

Dengue infection results into subclinical disease in majority of the cases and clinical disease in about 25% cases. Proportions of asymptomatic, mild cases and severe cases are highly variable in different areas. Differential diagnosis between clinically similar diseases caused by DENV, Chikungunya virus and other febrile illnesses is almost impossible in resource limited countries like India. Therefore clinical surveillance data which already suffers with tremendous reporting bias is inadequate to estimate true burden of disease. In such situations, properly designed seroprevalence studies may adequately quantify and characterize the extent of transmission.

Currently there is no effective drug for treatment of dengue. Sustained effective vector control has become impractical in developing countries. Therefore vaccination has become focus of attention in management of dengue. Several vaccines are in different phases of developments and clinical trials. The first live attenuated (recombinant) tetravalent dengue vaccine, Dengvaxia, produced by Sanofi Pasteur, has been licensed for use in some countries in Asia and Latin America. World Health Organization (WHO) Strategic Advisory Group of Experts (SAGE) recommends that countries consider introduction of this dengue vaccine only in populations where epidemiological data indicate a high burden of disease. In order to maximize public health impact and cost effectiveness, the populations to be targeted for vaccination, as measured by seroprevalence, should be approximately 70% or greater in the age group targeted for vaccination.[11] Seroprevalence typically increases with age, and countries may choose to target vaccination to the youngest age (9 years or older) for which seroprevalence exceeds the recommended 70% threshold.[12] Since such data is not available for most of the endemic places in India, well designed serosurveys are recommended to support decision making for vaccine introduction for public health as well as for conducting clinical trials with dengue vaccines.

In view of these concerns, a stratified serosurvey was conducted in Pune city, Maharashtra, India. Pune is fast growing city, chosen under Smart Cities Mission scheme of the Prime Minister of India for speedy and orderly infrastructure development. The city has been experiencing seasonal, annual dengue outbreaks. It is pertinent to generate data on epidemiological determinants including disease burden estimates for proper planning of dengue management.

## Methods

### Study area

Pune, the second largest city in the state of Maharashtra after Mumbai and the seventh most populous city in the country is situated 560 meters above sea level on the Deccan plateau. Pune is the administrative headquarters of Pune district and is one of the fastest growing cities in the Asia-Pacific region.

It lies between 18° 32" North latitude and 73° 51" East longitude. Pune is 149 kilometers, southeast of Mumbai by road. Average temperatures ranges between 19 to 33°C. Pune experiences three seasons: summer, monsoon, and winter. Typical summer months are from mid-March to June with maximum temperatures sometimes reaching 42°C. The monsoon lasts from June to October, with moderate rainfall and temperatures ranging from 22 to 28°C. Most of the 722 mm of annual rainfall in the city falls between June and September, and July is the wettest month of the year. In winter, the daytime temperature hovers around 26°C while night temperature is around 10–14°C, sometimes dropping to 5 to 6°C.

The population of the Pune city is 3,124,458 and Pune Urban Agglomeration is 5,057,709 as of the 2011 census.[13] Annual exponential growth rate of population was 2.08 per year (for 2001–2011), with birth rate of 19.3 live births per thousand of population per year.[13,14] In 2017, the estimated population of Pune is 3.99 million.[15] Pune city is divided into 5 administrative zones, having 15 administrative units called wards. Each ward has one or 2 clinics managed by Pune Municipal Corporation, many private clinics managed by General Practitioners, and some tertiary care hospitals.

### Study design

**Fig 1:**
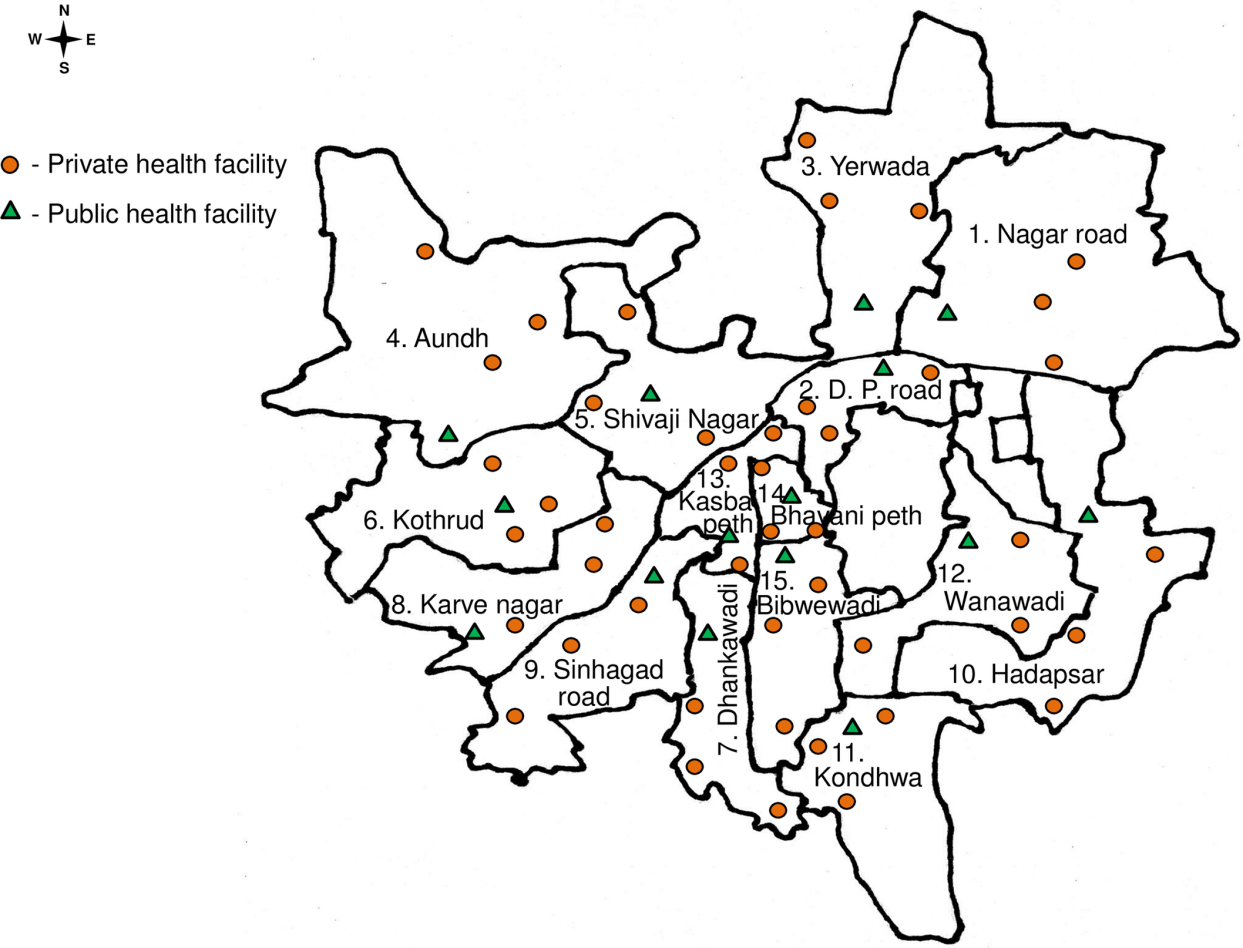
Geospatial distribution of the health facilities selected for conducting the sersurvey. Orange triangle symbol indicate PMC public health facility; round green symbols indicate private health facilities of general practitioners.

A cross sectional, stratified, facility based, multistage cluster sampling was conducted in Pune city between May 4 and June 27, 2017, following the principles of WHO guidelines.[12] The dengue season in this area is typically from July to December. The present survey was planned to capture activity of dengue from the previous 2016 dengue season. Medical clinics are the first contact point between febrile cases and health seeking facilities. In all 15 wards, a corporation clinic was chosen as first point for sampling. Additional 3 clinics of general practitioners were chosen in such a manner to provide fair representation to the ward. This ratio was based on assumption that about 25% of the primary healthcare in the city is provided by the corporation clinics and the rest by the private practitioners. Fig 1 shows approximate locations of the collection sites (health facility).

The data on dengue prevalence in Pune city were not available. However, dengue prevalence of 59% was reported from an urbanized village near Pune city.[16] Assuming that prevalence in Pune city will be higher than the adjoining urbanized village, for the purpose of sample size calculations we assumed 65% prevalence in Pune city. The minimum sample size of 1,396 participants was calculated under the assumption of 65% prevalence for dengue infection, α ± 5% error, Confidence level 95%. Accounting for the multistage sampling, the sample size considered a design effect of 4.0. Sample allocation to each ward and age groups was in proportion to the population of the ward and age group with respect to the Pune population. Allowing 5% additional samples to meet contingencies like insufficient sample, leakage and spoilage we targeted 1,465 samples. A team visited each health facility. Each non-febrile patient and/or the person accompanying them visiting the facility and resident of the same ward were invited to participate in the study. The willing persons were enrolled until the target sample collection was achieved for that site. Each enrolled person was requested to provide a blood sample following administration of ethical consent/assent approved by the Institutional Ethics Committee of the University.

### Sample collection

We collected blood samples from a total of 1,434 participants, 31 less than the original 1,465 sample target. About 5 mL blood was collected from each participants in anti-coagulant free vacutainer tubes (BD Bioscience) by trained phlebotomists and kept overnight at 4°C. Serum samples were separated by centrifugation at 3,000 rpm for 10 minutes and stored at -80°C.

### IgG antibody indirect ELISA

Each serum sample was tested for dengue IgG antibodies by ELISA using the commercial Panbio Dengue IgG Indirect ELISA kit (Panbio Diagnostics, Brisbane, Australia, Cat no. 01PE30) according to manufacturer’s instructions. The presence of detectable IgG antibodies indicates past exposure to dengue infection. Panbio units were calculated by dividing the sample absorbance by the cut-off value and then multiplying this value by 10. Samples were considered positive if Panbio units were >11, <9 Panbio units were considered negative and if Panbio units were between 9 to 11, samples were considered equivocal and retested to confirm the result.

### IgG antibody capture ELISA

An anti-dengue IgG-capture ELISA (Panbio Diagnostics, Brisbane, Australia, Cat no. 01PE10) was performed according to the manufacturer’s instructions. Anti-dengue IgG Panbio units were calculated by dividing the sample absorbance by the cut-off value and then multiplying this value by 10. Using this criteria, a value of >22 Panbio units was used to identify secondary infection. <18 Panbio units were considered negative for secondary infection and if Panbio units were between 18 to 22, samples were considered equivocal and retested to confirm the result for secondary infection.[17] High Panbio units are indicative of elevated levels of IgG antibodies which suggest that the patient has been recently exposed to dengue virus due to secondary infection.

### Dengue specific PRNT assay

As WHO recommends use of PRNT_90_ titers to minimize serum cross-reactivity with other dengue serotypes and flaviviruses prevalent in DENV endemic areas [12,18], we opted for PRNT_90_ method for this study. Due to resource constraint, we decided to process 120 indirect ELISA positive samples for PRNT. The selection of samples was based on Panbio units of IgG-positives (Indirect ELISA) arranged at the interval of 5 units and represented comparable proportions of total positives in each category.

We followed WHO guidelines for the PRNT_90_ test. However, since we were interested in assessing neutralizing antibodies (NAbs) against the currently circulating Indian strains, necessary modifications were made. The DENV strains used were DENV-1 (S19) (Accession no. MG053115), DENV-2 (S15) (Accession no. MG053142), DENV-3 (S111) (Accession no. MG053151) and DENV-4 (1028) (Accession no. MG272272) isolated during 2016 in Pune city [19]. The viruses actually used for PRNT were passaged 4–5 times, titrated using plaque assay and stored at -80°C at smaller aliquots. The test included two controls in duplicates; cell control without any virus or serum and virus control for different serotypes, without serum were used in the assay. For the test, early passage Vero cells (CCL-81, ATCC) were seeded at the density of 1 x 10^5^ cells/mL in Minimum Essential Medium (MEM) (GIBCO) with 10% Fetal Bovine Serum (FBS, GIBCO) in 24-well plate (1mL/well). The following day, serum samples (diluted 1:5 in MEM with 2% FBS) were heat inactivated at 56°C for 30 min and then serially diluted 4-fold in the same diluent in 96-well microtiter plates. Serially diluted serum samples were mixed with an equal volume i.e, 1:2 of diluted virus that gives 40–100 plaques/control well with each serotype. The final serum dilutions were 1:10 to 1:2560. After incubation for 1 hr at 37°C, 5% CO_2_ incubator, the medium was removed from 24-well plate and 100µl of each dilution of serum/virus mixture was added onto the cells in duplicate. The plates were then incubated for 1 hr for DENV-1, 2, 3 and 2 hr for DENV-4 at 37°C, 5% CO_2_ incubator to allow virus adsorption. After adsorption, 1ml of overlay media containing 1% Aquacide-II (Calbiochem) were added onto the cells and incubated for 3 days at 37°C, 5% CO_2_ incubator. Three days post infection, the overlay medium was discarded from the plates, and the cell monolayer was fixed with formalin for 30minutes at RT and permeabilized with 0.2% Triton X-100 in PBS for 5min. The cells were washed three times with PBS-T (0.02% Tween-20 in PBS) and stained with HB112 pan-flavivirus mouse monoclonal antibody (D1-4G2-4-15, ATCC) at 1:500 dilution in PBS for 2 hr. Cells were washed three times with PBS-T and incubated with goat anti-mouse IgG horseradish peroxidase (HRP) at 1:1500 dilution in PBS for 1 hr. After washing three times with PBS-T and two times with PBS, cells were stained with True Blue peroxidase substrate (KPL, Sera Care, MA, USA) and blue color staining of virus infected cells were counted as plaques. PRNT_90_ titer was calculated using NIH LID Statistical Web tool.[20] PRNT_90_ titer ≥ 1:10 to one dengue serotype at least was considered seropositive. A monotypic response was defined by the presence of NAbs against only one of the four DENV serotypes. A multitypic response was defined as a concomitant detection of NAbs against more than 1 serotype.

### Data analysis

Statistical analyses were performed using ‘R’ Version 3.4.1., Microsoft windows Excel 2010, SPSS v. 17.0 (SPSS Inc.,USA) and Graphpad Prism v.7.0 (Graphpad Software USA).[21] The logarithm (Log_10_) values of antibody titers of the serotypes were used for analysis and graphical representation. The statistical comparison of the means of the antibody titers of the serotypes was performed using analysis of variance (ANOVA). The association between the numbers of DENV serotypes (one, two and three simultaneous serotype infections) and mean age was performed using ANOVA with POST HOC Least significant difference (LSD) test, whereas their association with gender was tested through χ^2^ (chi-square) test for trend. Mann-Whitney U test was performed to check the association of PRNT_90_ titers against all 4 serotypes across different age groups.

#### Model selection

Catalytic models are used to estimate the dengue force of infection among dengue naïve children from age-specific seroprevalence data.[22,23] However, in these models the assumption that FOI is constant in time is inconsistent with current understanding of dengue epidemiology.[24] Due to non-availability of proper longitudinal clinical data and inherent limitation to capture unapparent infections in longitudinal clinical studies, actively collected well planned age stratified, cross sectional sero-survey data has been found more realistic for determination of FOI.[12]

We calculated force of infection (FOI), specifically to mean the annual risk of infection with any serotype among dengue-naive (seronegative) individuals.

For the simple catalytic model it is assumed that the constant force of infection acts upon members of susceptible population. This model predicts that the proportion of seronegative individuals declines with increasing age at a constant rate, λ, according to the relationship:

$$p_{a}=e^{-\lambda a}$$

where p_a_ is the proportion seronegative by age a years. Assuming that the risk of infection is constant over time, the force of infection parameter, λ, defines the rate at which seroconversion increases with age. The above model with assumption of constant force of infection can be expressed within a generalized linear modeling framework, such that:

$$‑\ln\left(p_{a}\right)=\lambda a$$ (model 1)

where λ can be estimated by maximum likelihood regression methods.

Another model was also fitted to allow the force of infection to vary with age.

$$‑\ln\;\left(\mathrm{pa}\right){=\lambda }_{1}a_{1}{+\lambda }_{2}a_{2}$$ (model 2)

a_1_ and a_2_ are linear terms of age for two different age groups. We estimated the force of infection parameters, with corresponding 95% confidence intervals. The likelihood ratio (LR) test was used to determine the level of statistical support for the model with age-varying force of infection over the model assuming a constant force of infection. The Akaike information criterion (AIC) was used to compare model fit, favoring the model with the lowest AIC value.

According to Ferguson et al., (1999) usually the proportion of seropositive cases increases with age, in which case the rate of increase with age can be interpreted as a measure of the intensity of the infection experienced by the population in the past.[23] The possibility that the age specific variation is due to mosquito biting pattern is considered insignificant in dengue transmission. It is reasonable to believe that rate of change in seropositivity with increasing age can also be considered as a function of time. Therefore in our analysis age is also taken as a time variable in some places for clarity of observations.

Following Tam et al., we used the term “Force of infection” specifically to mean the annual risk of infection with any DENV serotype among dengue seronegative individuals.[22] This is equivalent to the rate of seroconversion, and these terms were used interchangeably.

#### R_0_ estimation

R_0_ is the number of secondary infections generated by a primary case in a completely susceptible population. R_0_ gives insight into the level of control that is required to reduce incidence and eventually block transmission. R_0_ was computed using the method proposed by May and Anderson (1985) for growing populations. [25]

R_0_ = B/A’ where B is the reciprocal of the birth-rate and A = 1/ λ.

We assumed B to be 27.6/1000 in 2016, Pune urban Census data, Maharashtra. This method assumes that one can get up to 4 infections.

Grading of DENV transmission intensity in the population is based upon seroprevalence level among children 9 years of age (SP9). Transmission intensity is considered as very low (SP9 = 10%), low (SP9 = 30%), moderate (SP9 = 50%), high (SP9 = 70%), and very high (SP9 = 90%).[11,26]. We computed SP9 from the best fit catalytic model.

### Ethical consideration

Studies were conducted at Interactive Research School for Health Affairs (IRSHA), a constituent unit of Bharati Vidyapeeth (deemed to be University), Pune. The study was approved by the Institutional Ethics Committee (IEC/2017/04). Written consent/assent to participate in the study, reviewed and approved by the Ethics committee, was administered to each participant or to their legal guardian. All data were handled anonymously and confidentially.

## Results

### Demographic data

In this study, 1434 participants were recruited from 15 wards of Pune city. Of these, 723 (50.4%) were men and 711 (49.6%) women, 401(28.0%) were children ≤18 years and 1033 (72.0%) were adults >18 years. The age ranged from 1 month to 85 years with a mean of 31.2 years and a median of 29 years (Table 1).

**Table 1:** Number and proportion of participants tested positive by indirect IgG ELISA in Pune, India, 2017.

Variables	Number of participants	Numbers of participants positive	Positive Proportions	95% CI	
				Lower	Upper
Overall prevalence	1434	1163	0.811	0.790	0.831
Male	723	589	0.815	0.786	0.843
Female	711	574	0.807	0.777	0.836
Age groups in years					
0–3	51	11	0.216	0.103	0.329
4–6	64	24	0.375	0.256	0.494
7–9	73	35	0.479	0.365	0.594
10–12	101	60	0.594	0.498	0.69
13–15	68	48	0.706	0.598	0.814
16–18	44	34	0.773	0.649	0.897
19–20	50	40	0.800	0.686	0.914
21–30	296	256	0.86	0.826	0.904
31–40	265	242	0.913	0.879	0.947
41–50	179	174	0.972	0.948	0.996
51–60	125	123	0.984	0.962	1.006
61–70	76	74	0.974	0.938	1.01
>70	42	42	1.000	1.000	1.000
Wards (Zone numbers)					
Nagar Road (1)	108	91	0.843	0.774	0.912
Dhole Patil Rd (1)	80	73	0.913	0.851	0.975
Yerwada (1)	116	101	0.871	0.810	0.932
Aundh (2)	102	63	0.618	0.523	0.712
Shivaji Nagar (2)	87	64	0.736	0.643	0.829
Kothrud (2)	116	88	0.759	0.681	0.837
Dhankawadi (3)	105	80	0.762	0.681	0.843
Karve Nagar (3)	91	78	0.857	0.785	0.929
Sinhagad road (3)	117	87	0.744	0.665	0.823
Hadapsar (4)	113	94	0.832	0.763	0.901
Kondhwa (4)	68	52	0.765	0.664	0.866
Wanawadi (4)	79	75	0.949	0.900	0.998
Kasba Peth (5)	76	66	0.868	0.792	0.944
Bhavani peth (5)	88	78	0.886	0.820	0.952
Bibwewadi (5)	88	73	0.830	0.752	0.908

### Seroprevalence

**Fig 2:**
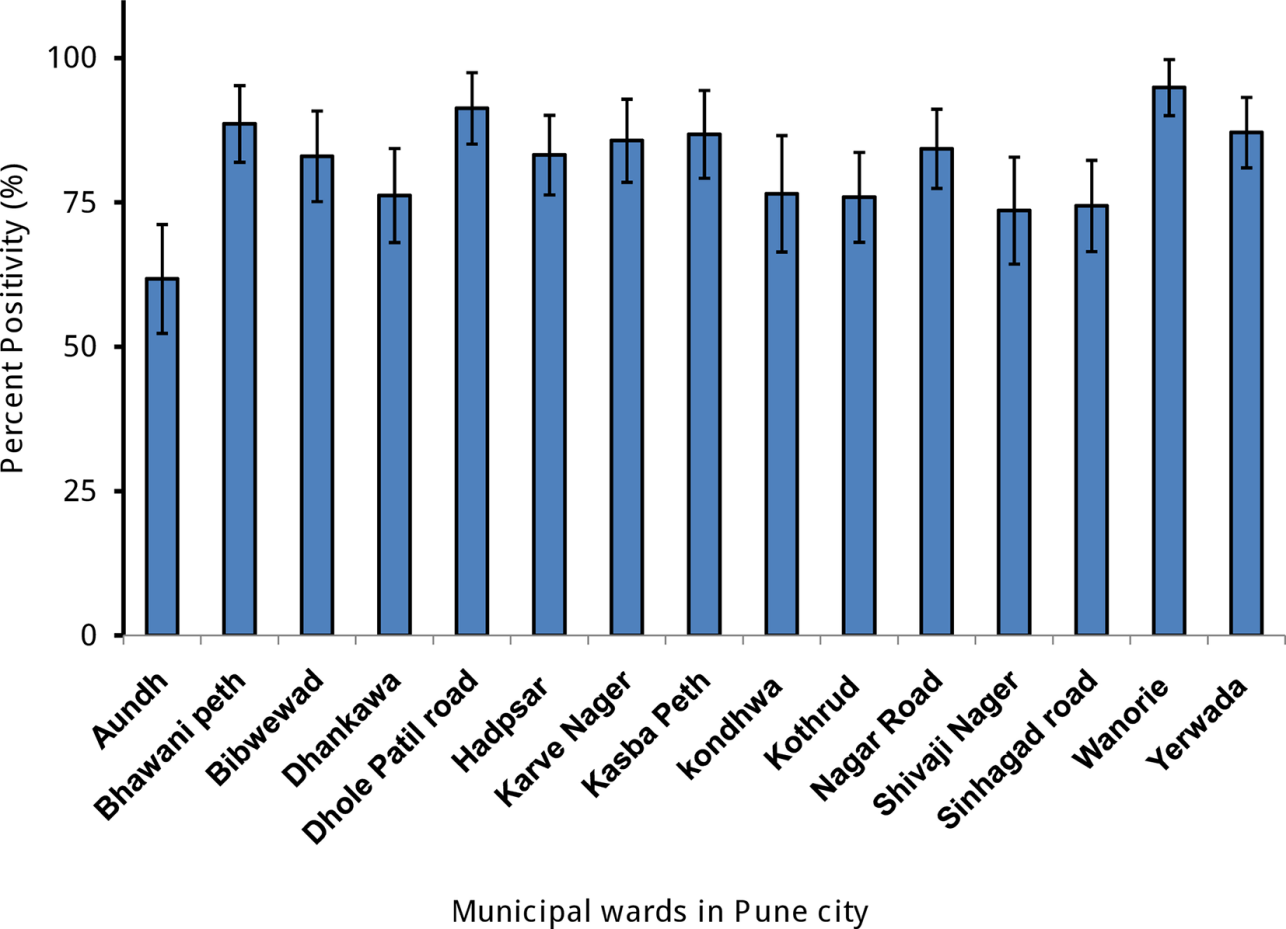
Percentage of participants tested positive by indirect ELISA in different municipal wards in Pune city. Error bars show 95% confidence intervals.

Ward-wise sample seropositive for anti-DENV IgG antibody by indirect IgG ELISA is presented in Fig 2. Overall percent seropositivity was 81%. The median age of seropositives was 33. The percent seropositivity between wards was significantly different (p< 0.001). The proportion of seroprevalence varied among the wards from moderate high in Aundh (61.8%) to very high in Wanawadi (94.9%).

The difference in percent seropositivity between males (81.5%) and females (80.7%) was not significant (p = 0.745). Similarly, there was no significant difference (p = 0.786) in percent seropositivity between the participants visiting GP clinics (80.96%) and Corporation clinics (82.12%). Only 92 of 1,205 seropositive individuals (7.6%) could remember having dengue in the past.

### Age stratified seroprevalence

**Fig 3:**
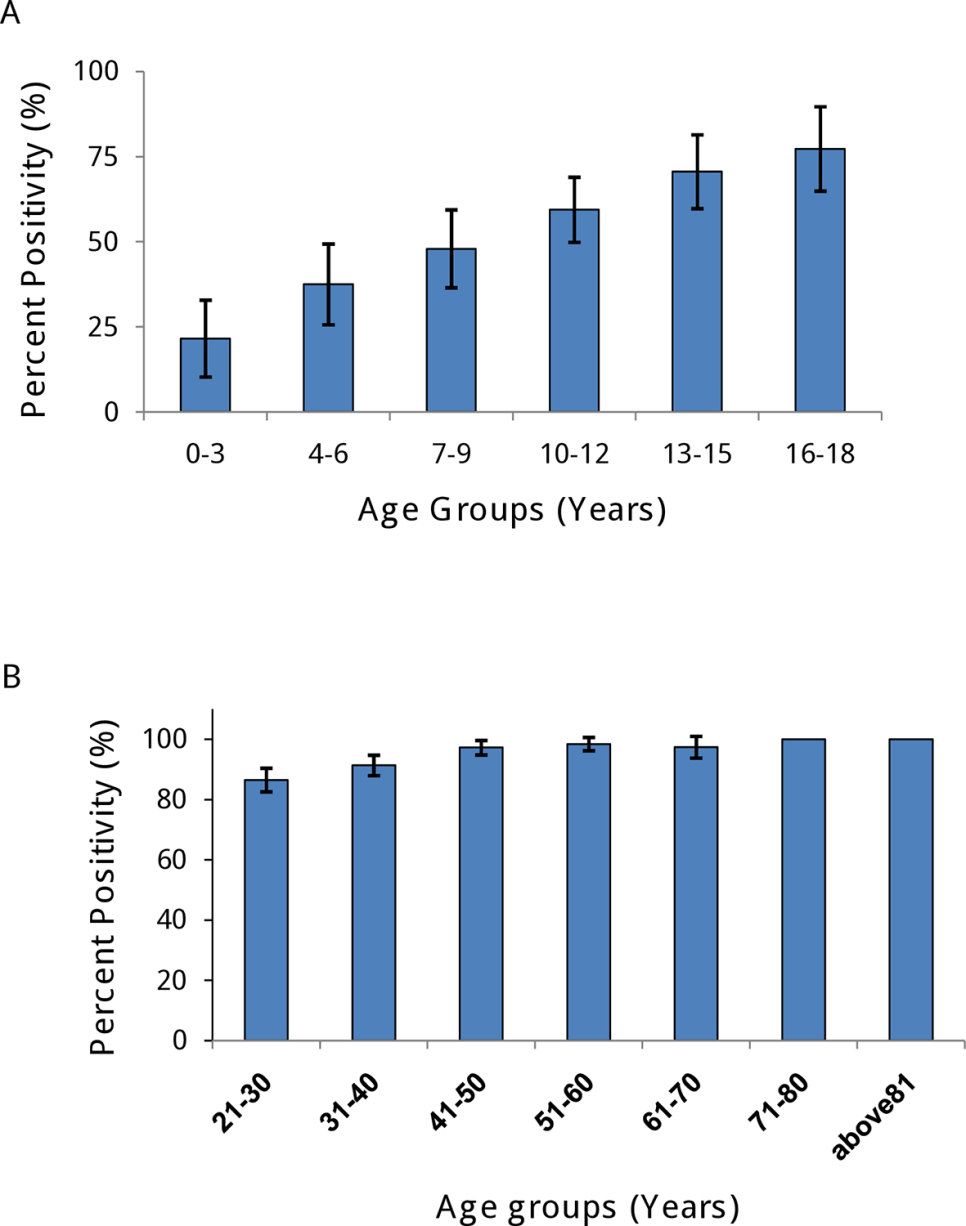
Percentage of participants tested positive by Indirect IgG ELISA in (A) age group ≤ 18 years, (B) adults in all study sites in Pune city. Error bars show 95% confidence intervals.

Distribution of seropositive samples in different age group is presented in Table 1. There was an increasing trend with age, from 21.6% among < 36 months group to 77.3% in age group 16–18 years. The positivity was significantly different (p<0.001) in different age groups in children ≤ 18 years but not significantly different in adults (Fig 3A and 3B). In adults > 70 yrs (n = 42) all the persons were seropositive. A third order linear polynomial model is best fit to the overall data (R^2^ = 0.97). Our estimated seroprevalence at 9 years age (SP9) was 54.17% (95% CI: 49.13% - 58.97%), which is classified as a low-to-moderate DENV transmission intensity.

### IgG capture ELISA

This test is designed to detect high levels of anti-DENV IgG antibodies indicative of a secondary infection. A total of 150 of 1,363 samples tested were positive (11.01%; 95% CI: 9.3%-12.6%). Overall seropositivity was highly variable between wards, ranging from 2.91% in Kondhwa to 20.95% in Hadapsar (S1 Table and S1 Fig).

**Fig 4:**
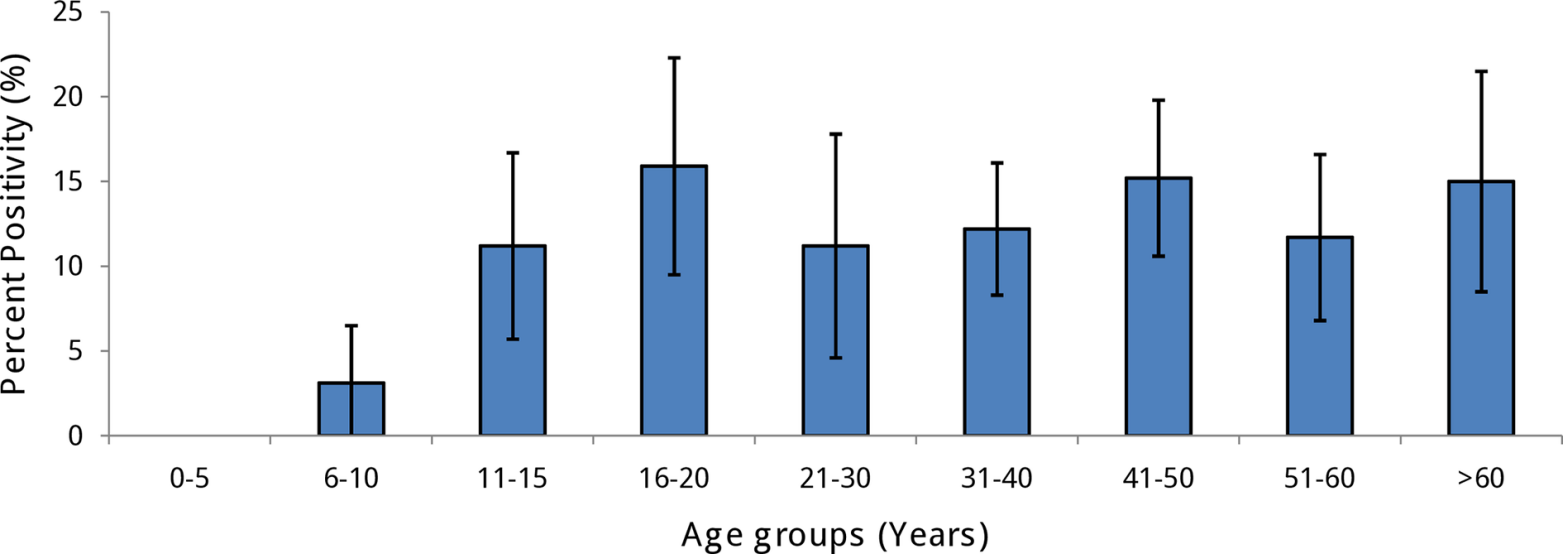
Percentage of participants tested positive by IgG Capture ELISA according to age groups in all study sites in Pune city. Error bars show 95% confidence intervals.

Only 4 of 229 children in age group ≤ 10 (1.7%) were seropositive suggesting a very low rate of secondary infection in young children. The seropositivity in older age groups varied between 11.2 and 15.9%. Overall distribution of positive proportions was non-linear suggesting age independent phenomenon **(**Fig 4).

### Plaque Reduction Neutralization Test (PRNT)

A total of 120 indirect IgG ELISA positive samples were tested for the presence of neutralizing antibodies by PRNT. Of these, 119 samples were confirmed to be seropositive via the presence of neutralizing antibodies and PRNT_90_ titers of ≥ 10. One sample had a PRNT_90_ titer of <10 against all 4 DENV serotypes and was considered seronegative (**Table 2)**. Over 69.2% samples were positive for DENV 1–4 followed by 11.7% samples which were positive for 3 serotypes, DENV2, DENV-3 and DENV-4. There was significant difference in the percent positivity for different serotypes (p<0.01).

**Table 2:** Distribution pattern of dengue serotype-specific neutralizing antibodies by PRNT.

Anti-dengue serotype-specific neutralizing antibodies	Frequency N = 120	Percent (%)	Mean age (95% CI)	Geometric Mean Titer (GMT) (Range)*			
				DENV1	DENV2	DENV3	DENV4
Not positive for any antibody	1	0.8	-	-	-	-	-
**Monotypic**							
DENV-1	0	-	-	-	-	-	-
DENV-2	5	4.2	27.8 (2.90–52.69)	-	74.26 (12–255)	-	-
DENV-3	1	0.8	35	-	-	729.10	-
DENV-4	2	1.7	26 (8.37–43.63)	-	-	-	154.21 (131.90–180.30)
**Subtotal**	8						
**Multitypic**							
DENV-1 and DENV-2	4	3.3	19.75 (6.35–33.14)	87.23 (19.00–267.50)	27.06 (13.50–59.30)	-	-
DENV-2 and DENV-4	4	3.3	33.25 (24.58–41.91)	-	113.87 (21.60–338.90)	-	60.28 (19.80–305.30)
DENV-2 and DENV-3	1	0.8	35	-	280	44.3	-
DENV-1, DENV-2 and DENV-3	2	1.7	37.5 (28.68–46.31)	38.87 (25.70–58.80)	63.71 (61.70–65.80)	31.93 (29.90–34.10)	-
DENV-1, DENV-2 and DENV-4	3	2.5	20.66 (5.59–35.72)	31.78 (18.50–66.50)	57.01 (43.40–73.50)	-	11.9 (1.40–117.10)
DENV-2, DENV-3 and DENV-4	14	11.7	38.57 (28.03–49.10)	-	227.43 (55.50–1645.50)	31.31 (10.20–129.20)	65.35 (10.00–1616.30)
DENV-1, DENV-2, DENV-3 and DENV-4	83	69.2	36.31 (32.42–40.19)	94.48 (10.50–3264.10)	516.63 (43.00–99167.90)	167.43 (14.20–2560)	97.75 (4.90–3264.10)
**Subtotal**	111						
**Total**	119						
Overall GMT (range)				89.135 (10.50–3264.10)	334.45 (12.00–99167.90)	128.60 (10.20–2560)	88.91 (1.40–1718.60)

*GMT was calculated for sera with PRNT_90_ titer ≥ 10

Percent PRNT positives for all DENV serotypes in different age groups are shown in Table 3. Amongst PRNT positives, DENV-2 was the most prevalent serotype across all age groups (94.4–100%). Only 5–6% individuals of age group up to 15 years were susceptible to DENV-2 and all individuals > 44 years of age were seropositive to this virus (Table 3). DENV-3 and DENV-4 follow age dependent linear distribution suggesting endemic nature of these serotypes for long duration but introduced late in comparison to DENV-2. DENV-1 was also prevalent across all the age groups in comparatively lower proportion and follows time independent distribution suggesting recent introduction. There is significant difference for percent positivity among different DENV serotype for age group 15 to 44 years (p = 0.006) and age group 60 years and above (p = 0.026) (Table 3). The sample size for PRNT was not enough for serotype specific model building for force of infection.

**Table 3:** Age group-wise dengue serotype specific neutralizing antibodies by PRNT in a subset of participants having antibody titers ≥10 (1/dil) against dengue viruses.

Age groups (n = total number of samples)	DENV-1 Number (%)	DENV-2 Number (%)	DENV-3 Number (%)	DENV-4 Number (%)	p-value
5–14 (n = 18)	14 (77.78)	17 (94.44)	11 (61.11)	12 (66.67)	0.1018*
15–44 (n = 70)	53 (75.71)	67 (95.71)	60 (85.71)	62 (88.57)	0.006*
45–59 (n = 14)	11 (78.57)	14 (100.00)	13 (92.86)	14 (100.00)	0.092*
≥ 60 (n = 17)	13 (76.47)	17 (100.00)	16 (94.12)	17 (94.12)	0.026*
Mean ± SD	31.28± 22.09	25.40± 13.35	33.94± 17.96	36.66± 18.29	0.279**

** ANOVA

* Chi-Square

**Fig 5:**
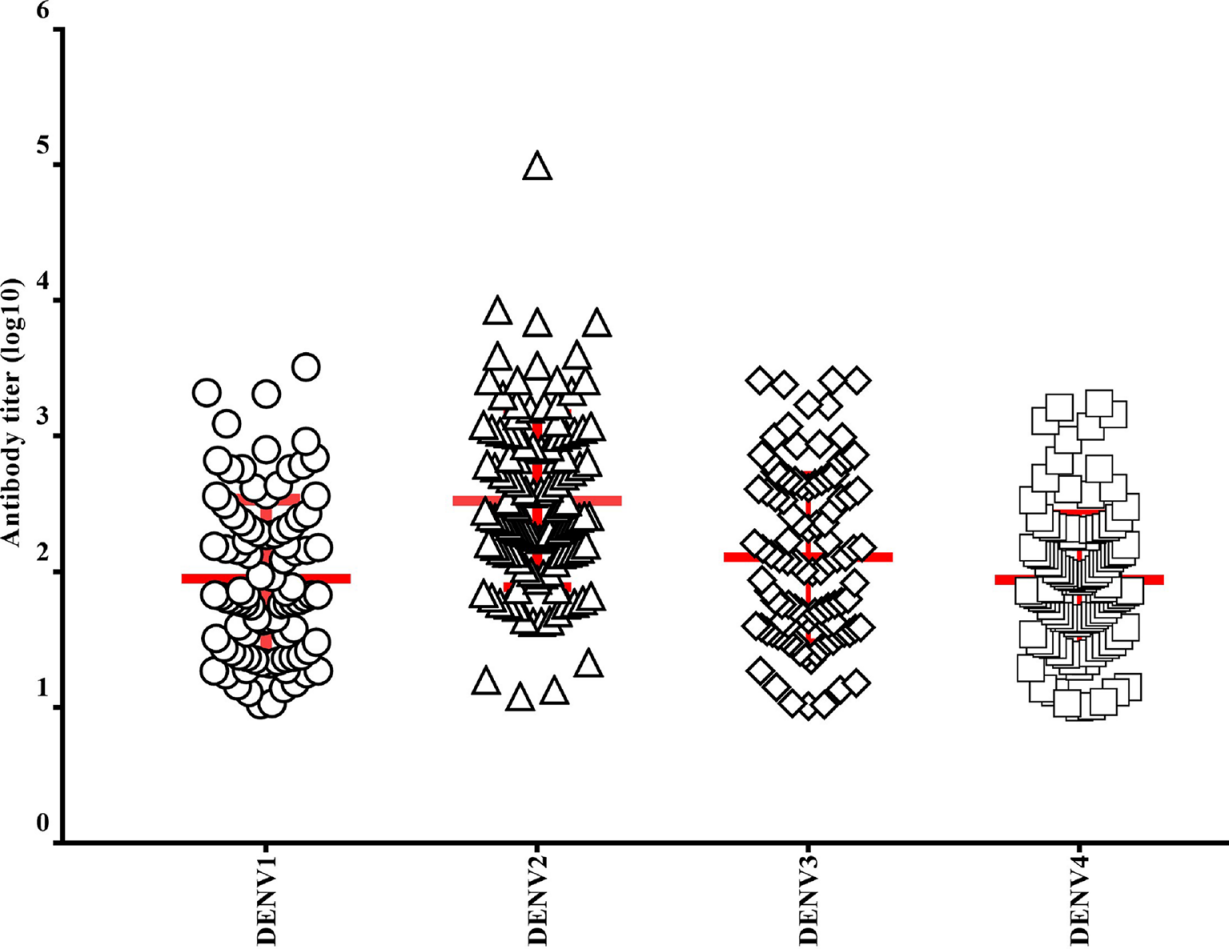
PRNT_90_ antibody titers (log_10_) in different dengue serotypes (Red line indicates mean titers with standard deviation).

Higher titers of neutralizing antibodies (log_10_ PRNT_90_) were detected in individuals infected with DENV-2 (2.524; 95% CI: 2.407–2.641) compared to other serotypes. The titer for DENV-4 was lowest among four serotypes (1.943; 95% CI: 1.844–2.041). There was significant difference between overall neutralizing antibody titer of the four serotypes (p<0.05; F = 23.568). Post hoc (LSD) test showed that this difference is because of neutralizing antibody titer of DENV-2 (2.524; 95% CI: 2.407–2.641) which was significantly higher than the titers of all other serotypes (p<0.05) and there is a significant difference between titer of DENV-3 (2.109; 95% CI: 1.987–2.231) and DENV-4 (1.943; 95% CI: 1.844–2.041) (Fig 5).

**Fig 6:**
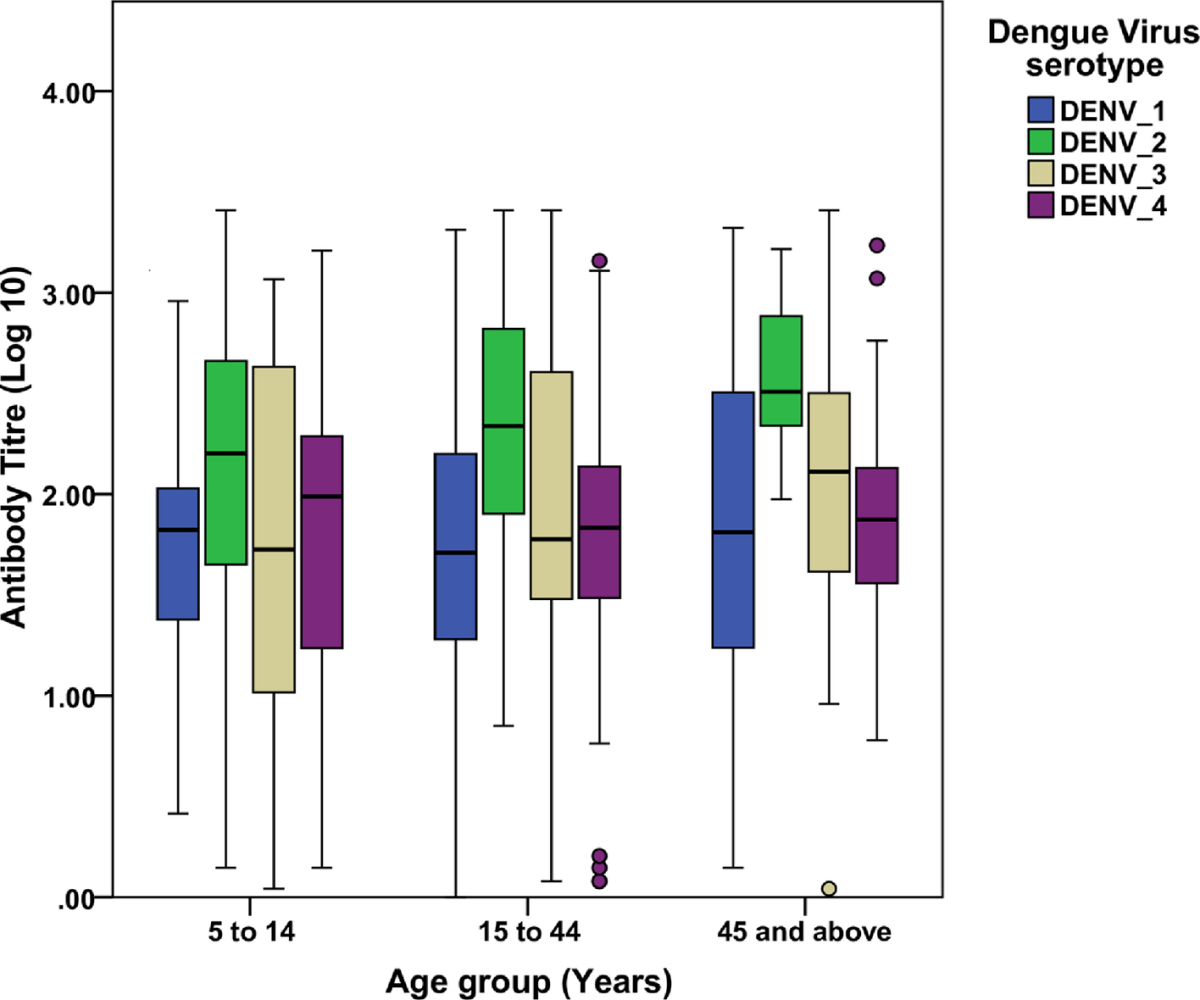
Age group specific PRNT_90_ titers against the four DENV serotypes. The error bars are 95% CI.

The titer of DENV-2 was highest across all age groups followed by DENV-3. In younger age groups, DENV-1 exhibited lower titer and in higher age groups DENV-4 showed lowest titer. However, differences in the titers across all age groups were not significant (Fig 6).

### Force of infection, basic reproductive number and burden of infection

**Fig 7:**
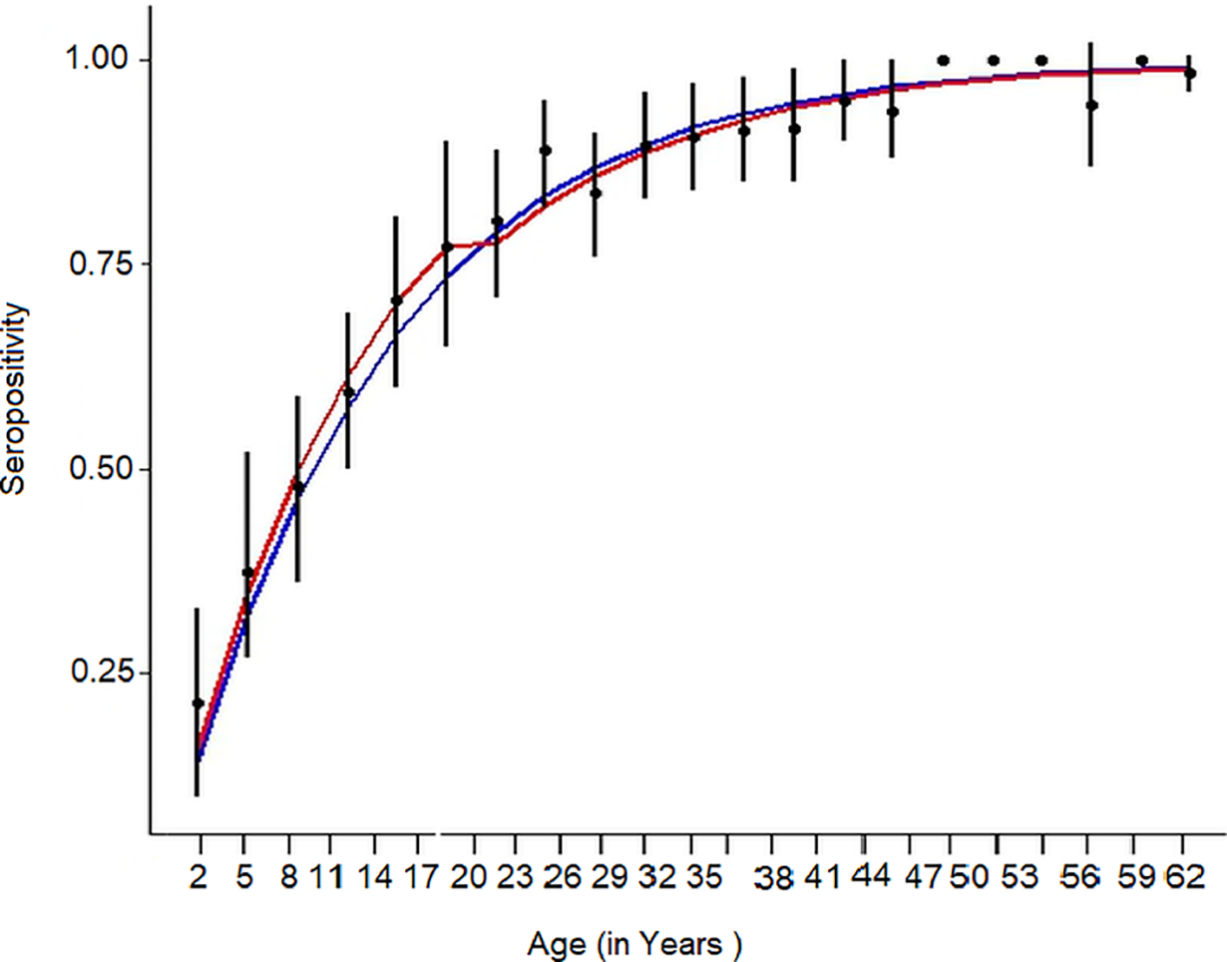
Observed prevalence by age (with 95% CIs) and model-predicted values. Data was aggregated into 3-year groups. Blue line: Model 1 (constant force of infection); Red line: Model 2 (different forces of infection above and below 18 years).

To estimate the transmission intensity of dengue, two catalytic models were fitted to the age-specific seroprevalence for indirect ELISA data, time constant (model A) and time varying (model B) forces of infection (Fig 7). As per model A, dengue naive children seroconverted at the rate of 7.81% per year (95% CI: 7.24%-8.43%). Under model B, annual rate of seroconversion was 8.68% (95% CI: 7.52%-9.95%) in younger population ≤ 18 years and 7.51% (95%CI: 6.87%-8.20%) in individuals > 18 years. The LR test showed non-significant association to favor any model B (p = 0.091) (Table 4). We also fitted model B with 15 and 12 years age break point without any significant difference in FOI. Thus the model B was consistent with a significantly higher force of infection during the period 2000–2016 (λ = 0.868; 95%CI: 0.752–0.099).

**Table 4:** Estimates of the force of infection (λ, summed across all serotypes) obtained from models fit to dengue age-specific sero-prevalence data.

Description	Estimate	95% CI			AIC*	p_value LR test**
		Lower	Upper	p_value		
Constant force of infection	0.07812	0.0724	0.0842	<0.001^#^	87.699	
Force of infection <18	0.08676	0.07518	0.0995	<0.001^#^	86.836	0.09062
Force of infection ≥18	0.07506	0.06872	0.0820	<0.001^#^		

* indicates Akaike information criterion

** indicates Likelihood ratio test

# p-value indicates that there is significant change in seropositivity with age.

Our estimated basic reproductive number (R_0_) for dengue in Pune is 4.23. These estimates assume endemic circulation of 4 serotypes and were derived using the FOI estimates and census data. The sample size was not enough to calculate FOI for individual wards. We pooled data by zone. Each zone consists of 3 adjoining wards. The mean R_0_ for Pune was 4.23 (95% CI: 3.58–4.87), lowest 3.41 in zone 3 and highest 5.25 in zone 1 (S2 Fig).

For estimation of the burden of disease, we have taken FOI as 8.68% for primary infection and 7.51% for secondary infections (Table 4). Accordingly in Pune city with estimated population of ~3.99 million in 2016, we estimate that this leads to approximately 65,800 (95% CI: 57009–75507) primary infections and 242,716 (95% CI: 222,032–265,016) secondary infections per year. Assuming that 69% immune population is positive for all 4 serotypes (Table 2), only 31% of secondary cases are likely to give rise to active dengue cases. Therefore, 75,242 secondary infections only are considered potentially secondary infections for estimation of dengue cases. The ratio between in-apparent and symptomatic dengue cases is highly variable, ranging from 1:1 to 3:1. Considering a ratio of 3:1, Pune city is burdened by about 47,000 symptomatic dengue cases each year.

We found 11.1% seropositivity in Capture IgG ELISA. This test is designed to detect high levels of anti-DENV IgG antibodies indicative of secondary infection. This translates to 358,741 secondary infections; 111,210 potential secondary cases and 59,000 dengue cases each year in Pune.

## Discussion

Dengue was first reported in India from Calcutta in 1912.[27] Now, it is a well established endemic disease in majority of Indian cities with occasional epidemics.[28,29] In Pune city, sporadic cases were reported in 1970s and 80s. Seasonal outbreaks have been recorded from 90s in different localities of the city with hemorrhagic involvement in some cases.[30] In spite of high prevalence of clinical disease, limited information is available on prevalence and incidence of the disease in India. Overall 81% IgG positivity by indirect ELISA with ~ 100% positivity in age groups > 45 years reported by us is much higher than 43% and 59% reported from 2 villages near Pune. In our study, seroprevalence of dengue was 50% in children of 6–10 years age group. In the same age group, high positivity is reported in Mumbai (80%), Delhi (60.2–66.5%), Wardha (69%), Bangalore (62%), Hyderabad (58%) and low positivity in Kalyani (23%).[31] The seropositivity of 79.3% in age group from 5–40 years is lower than 93% reported in Chennai.[32] Seropositivity of 11% by Capture ELISA and 81% by Indirect ELISA in our study is similar to the report from Hyderabad.[33] High seropositivity is also reported in Asian countries like Thailand, Bangladesh, Indonesia etc. [34–36]

Human population density is reported to be an important variable associated with a high historical incidence of dengue.[37–40] The level of seroprevalence seems to be also associated with the population size of the city. Small places like a village near Pune, population (2,621) and Kalyani in WB (population 100,575) reported low seroprevalence. Hyderabad and Pune similar in population pattern have nearly similar dengue prevalence; Chennai, Mumbai and Delhi, the metropolitan cities reported higher seroprevalence.

The lowest FOI and R_0_ for Indian subcontinent reported was based upon the data from Andamans island collected in 1988–89.[6,41] Population of this region was also very small on individual islands. In our study, only 7.6% participants could recall having the disease which is suggesting of a high frequency of unapparent infection or mild undifferentiated fever in agreement with other epidemiological studies.[42–45]

We estimated FOI, seroconversion rate, 8.68% in younger age group ≤ 18 years and 7.51% in older age groups. This is very different from 23% in Chennai. The reported seroconversion is highly variable in different places. In Sri Lanka, 8% seroconversion was reported in children ≤ 12 years age, 11 to 17% among children aged 2 to 15 years in Vietnam, 2.1 to7.9% in Thailand, 10% in Bangladesh, 13.1% primary infection per year in children in Indonesia, 17% in children aged 3 years in Salvador, Brazil.[22,32,35,46–52]

Our estimated R_0_ for dengue, 4.3 is lower than 5.3 estimated in Chennai and is comparable to estimates in hyperendemic settings in Thailand and Brazil.[6,53,54] As reported in other places, we also found significant heterogeneity between different wards.

In India, this is the first study to provide data on PRNT_90_, the test recommended by WHO for survey for neutralizing antibodies. Over 69% indirect ELISA positive samples were positive for all 4 serotypes followed by 11.7% positive for 3 serotypes, DENV-2, DENV-3 and DENV-4. DENV-2 was the most prevalent (94.4%) serotype across all age groups. This suggests widespread circulation of all the serotypes in Pune for quite some time. In another study, we reported active circulation of all the serotypes in Pune during 2016 dengue season.[19] Only other report from India, based on PRNT_50_ titers in children of 5–10 years age groups, reported overall positivity of 97.2% for at least one serotype, 79.7% for all four serotypes. DENV-1 was dominant serotype in Delhi; DENV-2 in Mumbai, Wardha, Bangalore and Hyderabad; DENV-3 in Kalyani. There is ample evidence that all 4 serotypes have been circulating in majority of the Asian countries.[55,56]

For analysis of neutralization tests, some investigators used PRNT_60_, others PRNT_50_ [31,51] making it difficult to compare the results of different studies. Following WHO recommendations,[12] we used PRNT_90_. In case of DENV-2, the multitypic response was positively associated with age because of the diversity of antibodies generated as a result of ongoing exposure. Highest neutralizing antibody titers observed for the DENV-2 suggests ongoing activity of this virus over the years and a multitypic response caused by the booster effect.[52,57,58]

One of the limitations of the present study is that PRNT was performed in a subset of individuals since it is expensive and laborious. Therefore the study population may not be true representative of the entire city. However, in spite of limitations our results provide valuable data on previous immunity at population level. For estimation of number of dengue cases, whether average seropositivity (11.1%) in capture ELISA for estimation of secondary cases in population can be extrapolated or not is an important issue. According to the manufacturer of the Panbio kit and others an IgG result of 22 Panbio units correlates with an HI titer of 1:1280, the cut-off used to distinguish between primary and secondary dengue infection.[59–61] Therefore, percent positivity in capture ELISA was used for estimation of secondary dengue under assumption that the high titered antibodies wane to below 22 Panbio units within a year and before next dengue season. However, there is a need to generate region specific data on decay pattern of these high titered antibodies in population. Further, in absence of testing for IgM antibody, possibility of primary infection in some cases cannot be ruled out.

Currently, the use and deployment of vector control as part of dengue outbreak response strategies is managed by public health in Pune city. It is highly unreliable and unsustainable due to limited resources and difficulties in management of human resources involved in vector control measures. There is no impact assessment in place for such a measure. There is also growing evidence that vector control is not a logical solution for control of dengue in large cities.[62] In the absence of specific drugs and limited usefulness of vector control measures, suitable vaccines are eagerly awaited.[63]

Dengvaxia, a live attenuated (recombinant) tetravalent vaccine is a licensed vaccine for dengue in several countries for children 9 years of age or older living in DENV endemic areas having high endemicity among 9 year-olds. Children who are seronegative at the time of first vaccination may be primed for future risk of severe dengue illness in areas of low to moderate (SP9 = 30%-50%) and even moderate to high (SP9 = 50%-70%) endemicity.[11] Therefore it was suggested that average seropositivity of 70% may be minimum requirement for introduction of the vaccine because of variability from locality to locality. With estimated average SP9 = 54% in present study. This vaccine is not suitable for Pune at this stage for the specified age group ≥ 9.

It has been well-documented that passive surveillance involving case notifications does not accurately reflect the burden of dengue in most of locations. Cohort studies in different provinces of Thailand and in Nicaragua had revealed higher numbers of prospectively determined dengue incidences as compared with national reported figures, with a discrepancy of 8 to 21.3-folds.[64–66] According to Shepard et. al. (2014) disease burden of dengue in India is 282 times the reported number per year, substantially more than captured by officially reported cases.[9] In this study, we estimated 47,000 to 59,000 cases per year in Pune city alone. As per official government report, only 6,792 cases of dengue were reported from whole of the Maharashtra in 2016. Therefore, it is strongly recommended that for a disease like dengue, serosurveys should be conducted periodically. It could shed light on the true dengue infections in the population and can be a good tool to monitor impact of interventions at population level.

## Supporting information

S1 Checklist STROBE checklist. (DOCX)

S1 Table Description of the proportions of participants positive by capture IgG ELISA by wards and age_groups in Pune city. (PDF)

S1 Fig Ward-wise seropositivity by capture IgG ELISA in Pune city. (TIF)

S2 Fig Histogram showing the distribution of zone specific R_0_ estimates from the data. (TIF)
