# Supplementary material for: Stratified sero-prevalence revealed overall high disease burden of dengue but suboptimal immunity in younger age groups in Pune, India
Source: PLoS Negl Trop Dis. 2018 Aug 6;12(8):e0006657. doi: 10.1371/journal.pntd.0006657 (PMC6095695; doi:10.1371/journal.pntd.0006657)
Supplement: S1 Checklist — (DOCX) [file pntd.0006657.s001.docx]

S1 Checklist: STROBE Checklist

STROBE Statement— a ***cross-sectional study***

|  | **Item No** | **Recommendation** |
| --- | --- | --- |
| **Title and abstract** | 1 | Indicate the study’s design with a commonly used term in the title or the abstract  [Abstract: methodology-paragraph 1] |
|  |  | Provide in the abstract an informative and balanced summary of what was done and what was found  [Abstract: methodology, Findings] |
| **Introduction** | | |
| Background/rationale | 2 | Explain the scientific background and rationale for the investigation being reported  [Introduction, first 4 paragraph] |
| Objectives | 3 | State specific objectives, including any prespecified hypotheses  [Introduction, last paragraph] |
| **Methods** | | |
| Study design | 4 | Present key elements of study design early in the paper  [Study design, line 129] |
| Setting | 5 | Describe the setting, locations, and relevant dates, including periods of recruitment, exposure, follow-up, and data collection  [Study area, Paragraph 1; study design, first paragraph; sample collection] |
| Participants | 6 | Give the eligibility criteria, and the sources and methods of selection of participants  [Study design, last paragraph] |
| Variables | 7 | Clearly define all outcomes, exposures, predictors, potential confounders, and effect modifiers. Give diagnostic criteria, if applicable  [IgG antibody indirect ELISA; IgG antibody Capture ELISA; dengue specific PRNT_90_ Tests] |
| Data sources/ measurement | 8* | For each variable of interest, give sources of data and details of methods of assessment (measurement). Describe comparability of assessment methods if there is more than one group  [IgG antibody indirect ELISA; IgG antibody Capture ELISA; dengue specific PRNT_90_ Tests] |
| Bias | 9 | Describe any efforts to address potential sources of bias |
| Study size | 10 | Explain how the study size was arrived at  [Study design, Paragraph 2] |
| Quantitative variables | 11 | Explain how quantitative variables were handled in the analyses. If applicable, describe which groupings were chosen and why  [Data analysis; model selection] |
| Statistical methods | 12 | Describe all statistical methods, including those used to control for confounding  [Data analysis] |
|  |  | (*b*) Describe any methods used to examine subgroups and interactions [None] |
|  |  | (*c*) Explain how missing data were addressed [Not applicable] |
|  |  | (*d*) If applicable, describe analytical methods taking account of sampling strategy |
|  |  | (*e*) Describe any sensitivity analyses |
| **Results** | | |
| Participants | 13* | 1. Report numbers of individuals at each stage of study—eg numbers potentially eligible, examined for eligibility, confirmed eligible, included in the study, completing follow-up, and analysed   [Results, Table 1; SI Table 1] |
|  |  | (b) Give reasons for non-participation at each stage |
|  |  | (c) Consider use of a flow diagram |
| Descriptive data | 14* | 1. Give characteristics of study participants (eg demographic, clinical, social) and information on exposures and potential confounders   [Results, Table 1; SI Table 1] |
|  |  | (b) Indicate number of participants with missing data for each variable of interest |
| Outcome data | 15* | Report numbers of outcome events or summary measures  [Results, seroprevalence] |
| Main results | 16 | Give unadjusted estimates and, if applicable, confounder-adjusted estimates and their precision (eg, 95% confidence interval). Make clear which confounders were adjusted for and why they were included  [ Results] |
|  |  | (*b*) Report category boundaries when continuous variables were categorized |
|  |  | (*c*) If relevant, consider translating estimates of relative risk into absolute risk for a meaningful time period |
| Other analyses | 17 | Report other analyses done—eg analyses of subgroups and interactions, and sensitivity analyses  [Results: Force of Infection, table 4] |
| **Discussion** | | |
| Key results | 18 | Summarise key results with reference to study objectives  [Discussion, paragraph 1] |
| Limitations | 19 | Discuss limitations of the study, taking into account sources of potential bias or imprecision. Discuss both direction and magnitude of any potential bias  [Discussions: paragraphs 4,5] |
| Interpretation | 20 | Give a cautious overall interpretation of results considering objectives, limitations, multiplicity of analyses, results from similar studies, and other relevant evidence  [Discussion, last paragraph] |
| Generalisability | 21 | Discuss the generalisability (external validity) of the study results  [Discussion, last 2 paragraphs] |
| **Other information** | | |
| Funding | 22 | Give the source of funding and the role of the funders for the present study and, if applicable, for the original study on which the present article is based  [Financial disclosure] |

*Give information separately for exposed and unexposed groups.
