# Supplementary material for: Stratified sero-prevalence revealed overall high disease burden of dengue but suboptimal immunity in younger age groups in Pune, India
Source: PLoS Negl Trop Dis. 2018 Aug 6;12(8):e0006657. doi: 10.1371/journal.pntd.0006657 (PMC6095695; doi:10.1371/journal.pntd.0006657)
Supplement: S1 Table — (PDF) [file pntd.0006657.s002.PDF]

S1 Table. Description of the proportions of participants with antibody titres≥ 22 units (1/dil) against dengue viruses by wards and age_groups by Capture ELISA in Pune city.

**Pune municipal ward name**	Capture Elisa			
	N	Positive	Positive proportion	(95%CI)
Aundh	98	5	0.051	(0.007 - 0.094)
Bhavani Peth	78	13	0.167	(0.083 - 0.249)
Bibwewadi	84	8	0.095	(0.032 - 0.158)
Dhankawadi	101	6	0.059	(0.013 - 0.105)
Dhole Patil Road	74	7	0.094	(0.027 - 0.161)
Hadapsar	105	22	0.209	(0.131 - 0.287)
Karve Nagar	87	11	0.126	(0.056 - 0.196)
Kasba Peth	70	6	0.085	(0.02 - 0.151)
Kondhwa	68	2	0.029	(0.0 - 0.069)
Kothrud	112	12	0.107	(0.049 - 0.164)
Nagar Road	101	7	0.069	(0.019 - 0.118)
Shivaji Nagar	83	10	0.12	(0.05 - 0.19)
Sinhagad Road	115	14	0.121	(0.061 - 0.181)
Wanawadi	75	10	0.133	(0.056 - 0.21)
Yerwada	112	17	0.151	(0.085 - 0.218)
**Age group**				
0-5	101	0	0	
6-10	128	4	0.031	(0.00 - 0.065)
11-15	124	14	0.112	(0.058 - 0.167)

16-20	88	14	0.159	(0.094 - 0.223)
21-30	276	31	0.112	(0.046 - 0.178)
31-40	244	30	0.122	(0.084 - 0.161)
41-50	170	26	0.152	(0.107 - 0.198)
51-60	119	14	0.117	(0.069 -0.166)
>60	113	17	0.150	(0.086 - 0.214)
Total	1363	150	0.110	(0.052 - 0.167)
