# Supplementary figures and images for: Stratified sero-prevalence revealed overall high disease burden of dengue but suboptimal immunity in younger age groups in Pune, India
Source: PLoS Negl Trop Dis. 2018 Aug 6;12(8):e0006657. doi: 10.1371/journal.pntd.0006657 (PMC6095695; doi:10.1371/journal.pntd.0006657)

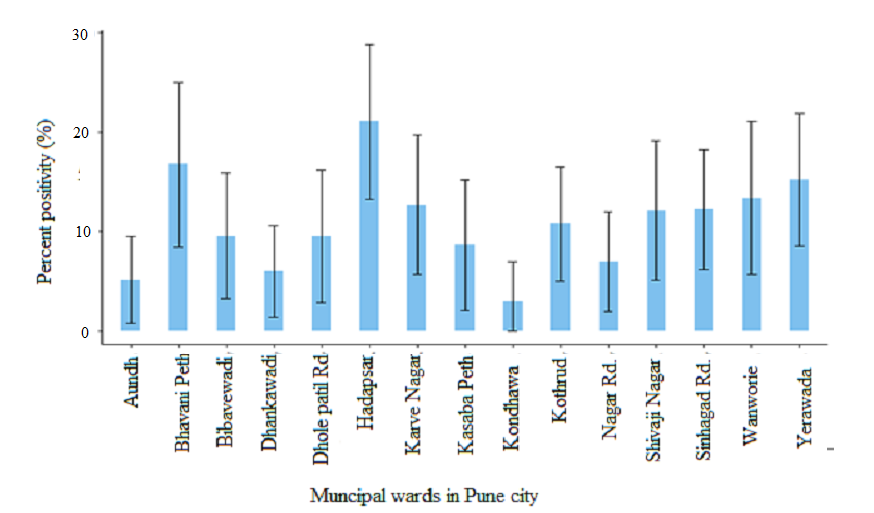

Supplement: S1 Fig — (TIF) [file pntd.0006657.s003.tif]

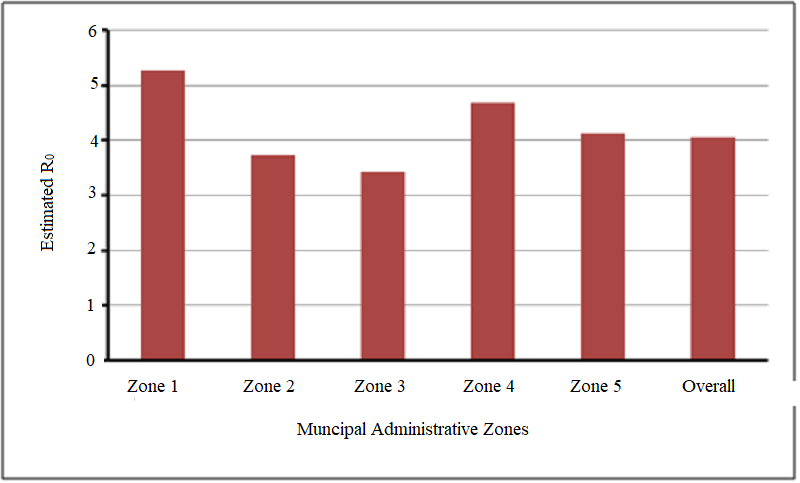

Supplement: S2 Fig — (TIF) [file pntd.0006657.s004.tif]
